# PROCESS EVALUATION OF THE RESIDENTIAL CARE TRANSITION MODULE

**DOI:** 10.1093/geroni/igae098.1256

**Published:** 2024-12-31

**Authors:** Dana Urbanski, Robyn Birkeland, Katie Louwagie, Dionne Bailey, Zachary Baker, Allison Gustavson, Lauren Mitchell, Joseph Gaugler

**Affiliations:** University of Tennessee Health Science Center, Knoxville, Tennessee, United States; University of Minnesota, Minneapolis, Minnesota, United States; University of Minnesota, Minneapolis, Minnesota, United States; University of Minnesota, Minneapolis, Minnesota, United States; Arizona State University, Phoenix, Arizona, United States; Minneapolis Veterans Affairs Health Care System, Minneapolis, Minnesota, United States; Emmanuel College, Boston, Massachusetts, United States; University of Minnesota, Minneapolis, Minnesota, United States

## Abstract

Although still relatively uncommon in dementia care science and gerontology, process evaluations can offer substantial insight when considering the dissemination and implementation of evidence-based innovations, particularly when utilizing various types of data to examine causal assumptions of an intervention as well as pre-specified contextual moderators or mediators that may influence intervention effects. This process evaluation leveraged the extensive quantitative and qualitative data collected during the 12-month, mixed methods evaluation of the Residential Care Transition Module (RCTM), a psychoeducational telehealth (via phone or video conference) intervention. Of 120 treatment participants, 107 completed the six session intervention. Session duration averaged 81 minutes (range 18-130 minutes, SD=20.9). The 13 participants who did not finish the intervention were bereaved during the intervention. As such, intervention delivery was discontinued. One hundred and fourteen treatment group participants engaged in varied amounts and means of ad hoc communications throughout their time in the study, including 9 of the 13 aforementioned bereaved participants who chose to continue to work with their transition coach after their relative passed. Eighty-one of the 114 participants engaged in ad hoc coaching sessions via phone or video conference, averaging six ad hoc sessions (range 1-38 sessions, SD=5.7). Ad hoc session duration averaged 41 minutes (range 5-120 minutes, SD=18.4). Emotional support was the most frequently delivered content, occurring in 91% of intervention sessions and 53% of ad hoc contacts. Participants overwhelmingly valued the RCTM program via a treatment review checklist administered quarterly, indicating high perceptions of utility, acceptability, and satisfaction with the RCTM.

